# The Role of Phytonutrient Kaempferol in the Prevention of Gastrointestinal Cancers: Recent Trends and Future Perspectives

**DOI:** 10.3390/cancers16091711

**Published:** 2024-04-27

**Authors:** Tejveer Singh, Deepika Sharma, Rishabh Sharma, Hardeep Singh Tuli, Shafiul Haque, Seema Ramniwas, Darin Mansor Mathkor, Vikas Yadav

**Affiliations:** 1 Translational Oncology Laboratory, Department of Zoology, Hansraj College, Delhi University, New Delhi 110007, India; deepika231098@gmail.com (D.S.); bioamicos@gmail.com (R.S.); 2 Division of Cyclotron and Radiopharmaceutical Sciences, Institute of Nuclear Medicine and Allied Sciences-Defence Research and Development Organization, (INMAS-DRDO) New Delhi, Delhi 110054, India; 3 Amity Stem Cell Institute, Amity Medical School, Amity University, Gurugram 122412, India; 4 Department of Bio-Sciences & Technology, Maharishi Markandeshwar (Deemed to Be University), Mullana, Ambala 133207, India; hardeep.biotech@gmail.com; 5 Research and Scientific Studies Unit, College of Nursing and Allied Health Sciences, Jazan University, Jazan 45142, Saudi Arabia; shafiul.haque@hotmail.com (S.H.); darin.mathkor@gmail.com (D.M.M.); 6 Gilbert and Rose-Marie Chagoury School of Medicine, Lebanese American University, Beirut 11022801, Lebanon; 7 University Centre for Research & Development, University Institute of Pharmaceutical Sciences, Chandigarh University, Gharuan, Mohali 140413, India; seema.ramniwas@gmail.com; 8 Department of Translational Medicine, Clinical Research Centre, Skåne University Hospital, Lund University, SE-20213 Malmö, Sweden

**Keywords:** kaempferol, gastrointestinal cancer, nanotechnology, liver cancer, pancreatic cancer, colorectal cancer, esophageal cancer, gastric cancer, apoptosis, cell cycle arrest

## Abstract

**Simple Summary:**

Kaempferol (3,5,7-trihydroxy-2-(4-hydroxyphenyl)-4H-chromen-4-one), a flavonoid, is richly found in fruits and vegetables. Kaempferol has been proven to reduce tumor cell growth by modulating several pathways and molecular mechanisms, such as causing G0/G1 phase arrest in esophageal cancer, reducing G2/M cell cycle proteins in gastric cancer, inducing apoptosis by Akt/mTOR pathway in pancreatic cancer, causing cell cycle arrest (especially HT-29 human colon cancer cells), and suppressing cell growth by PI3K/mTOR/MMP signaling pathways in liver cancer. The application of nanotechnology has been shown to enhance the efficacy of kaempferol against gastrointestinal cancer. Mechanistic studies showed kaempferol-conjugated nanoparticles inducing oxidative stress-mediated apoptosis and cell cycle arrest in liver cancer cells, potentially leading to anti-cancer effects. Notably, kaempferol-conjugated gold nanoclusters have also shown efficacy in reducing tumor volume *in vivo*. However, there is a lack of research specifically focused on gastrointestinal cancers, highlighting the need for further exploration in this area.

**Abstract:**

In recent years, kaempferol, a natural flavonoid present in various fruits and vegetables, has received significant attention in gastrointestinal cancer research due to its varied therapeutic effects. Kaempferol has been proven to alter several molecular mechanisms and pathways, such as the PI3/Akt, mTOR, and Erk/MAPK pathway involved in cancer progression, showing its inhibitory effects on cell proliferation, survival, angiogenesis, metastasis, and migration. Kaempferol is processed in the liver and small intestine, but limited bioavailability has been a major concern in the clinical implications of kaempferol. Nano formulations have been proven to enhance kaempferol’s efficacy in cancer prevention. The synergy of nanotechnology and kaempferol has shown promising results in *in vitro* studies, highlighting the importance for more *in vivo* research and clinical trials to determine safety and efficacy. This review aims to focus on the role of kaempferol in various types of gastrointestinal cancer and how the combination of kaempferol with nanotechnology helps in improving therapeutic efficacy in cancer treatment.

## 1. Introduction

The second leading cause of cancer-related death worldwide is gastrointestinal cancer (GI) [1]. Affecting the entire gastrointestinal tract and its associated organs, GI cancer encompasses various types, such as esophageal, colon, pancreatic, stomach (gastric), and liver cancer [2]. Symptoms and risk factors differ depending on the type of cancer. Individuals diagnosed with esophageal cancer may encounter challenges in swallowing, while those with stomach cancer might undergo symptoms resembling ulcers, such as indigestion, reduced appetite, bloating, and pain [3,4,5,6,7]. Continuous stomach acid problems, a diet high in salty and smoked foods, smoking, and a family history of the disease are all risk factors for stomach cancer [8]. Early identification is critical for efficient treatment, and gastrointestinal malignancies are identified using a variety of diagnostic procedures.

The type and stage of cancer determine which therapeutic measures are used. Surgical techniques, radiation therapy, chemotherapy, and targeted therapy are among the available treatment approaches, but these comes with numerous side effects, such as the fact that these therapies can damage the surrounding healthy cells [9,10]. Radiation therapy can cause long-term side effects to the brain, spinal cord, and nerves. Surgery may also result in side effects such as tissue scarring, pain, intestinal problems, and chronic diarrhea. Chemotherapy can lead to side effects like neutropenia, lymphedema, hair loss, nausea and vomiting, and blood clots [11]. Phytochemicals, which are bio-active compounds found in several fruits, cereals, and vegetables, such as apples, cherries, berries, kale, broccoli, spinach, tomatoes, and leafy vegetables have been examined for their possible role in disease prevention, particularly in gastrointestinal (GI) cancer [12]. Phytochemicals have fewer and less severe side effects as compared to conventional cancer treatments [13,14]. They have been shown to improve cancer prognosis through a variety of biological processes that increase apoptosis and prevent cancer growth [12]. Furthermore, phytochemicals have been shown to have strong anticancer effects, triggering apoptosis and autophagy, and decreasing tumor cell resistance to chemotherapeutic drugs in gastric cancer [15,16,17,18,19,20,21]. Some phytochemicals have been examined for their potential impact on GI cancer, including quercetin, ellagic acid, allicin, curcumins, and others [12,15,22,23,24].

**Figure 1:**
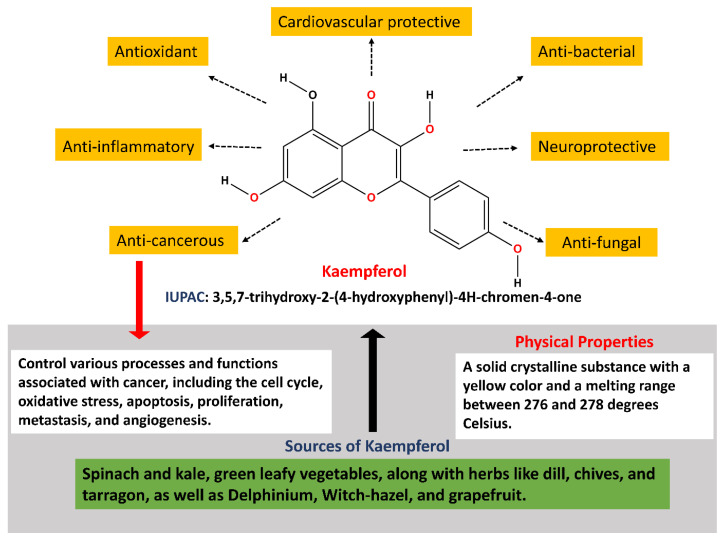
Diverse functions of kaempferol.

Kaempferol, a natural flavonoid having various functions (Figure 1), has interestingly shown potential in lowering GI malignancies, particularly colorectal and stomach cancers [25]. It has been shown in research studies that kaempferol reduces cancer cell proliferation, causes apoptosis, and blocks the cell cycle at the G2/M phase in colorectal and gastric cancer cell lines. Furthermore, kaempferol has shown its ability to overcome 5-fluorouracil resistance, indicating its usefulness as a chemotherapeutic drug. It has also been proved to affect several signaling pathways that are related with the progression and initiation of GI cancers [26,27,28]. This review will address the significance of kaempferol in preventing various GI malignancies.

## 2. Chemistry and Pharmacokinetics of Kaempferol

**Figure 2:**
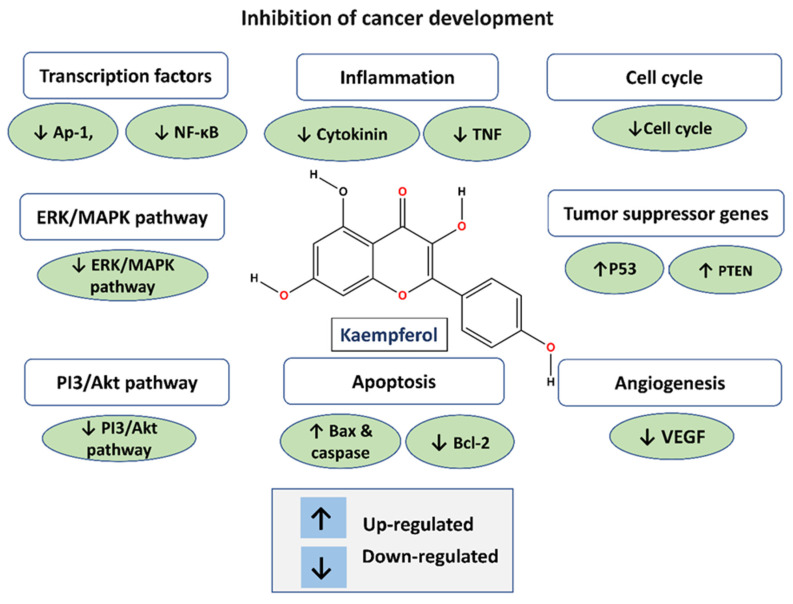
Anti-cancer function of kaempferol.

Kaempferol (3,5,7-trihydroxy-2-(4-hydroxyphenyl)-4H-chromen-4-one) is a polyphenol, a flavonoid that is richly found in fruits and vegetables. Kaempferol possesses a diphenylpropane structure, which contributes to its hydrophobic characteristics. Its synthesis begins with the condensation of 4-coumaroyl-CoA and three malonyl-CoA molecules, catalyzed by chalcone synthase, yielding naringenin chalcone. Subsequently, under the action of chalcone isomerase, naringenin chalcone is converted into naringenin, a flavanone. Then, through the enzymatic activity of flavanone 3-dioxygenase, a hydroxyl group is added to naringenin at the C3 position, resulting in dihydrokaempferol. Finally, the formation of kaempferol is accomplished by the introduction of a double bond at the C2–C3 position in the dihydrokaempferol skeleton, mediated by flavonol synthase (Figure 2) [29,30,31]. Kaempferol is primarily found in its conjugated form in both plasma and urine [32]. Kaempferol is frequently consumed in the form of a glycoside. Glycosides are compounds composed of a sugar molecule attached to a non-sugar molecule, referred to as an aglycone [33,34]. The colon’s flora helps in metabolizing kaempferol glycoside into aglycones, which is further metabolized and absorbed into systemic circulation [35]. This suggests that the metabolism of kaempferol involves its conjugation with other molecules, likely to enhance its solubility and facilitate its excretion from the body. Such conjugation processes play a crucial role in the pharmacokinetics and elimination of kaempferol from the systemic circulation, shaping its bioavailability and potential physiological effects [36].

Kaempferol, like other flavonoids, is recognized for its anti-inflammatory and antioxidant properties. Because of its antioxidant and anti-inflammatory qualities, kaempferol has been intensively researched for its possible significance in the prevention of gastrointestinal malignancies [37,38,39,40,41]. Kaempferol has been demonstrated to have various modes of action that contribute to its potential as a preventative agent against gastrointestinal malignancies, in addition to its antioxidant and anti-inflammatory characteristics [35]. For example, it has been proven to limit cancer cell development, cause apoptosis (programmed cell death), suppress tumor angiogenesis (the construction of blood vessels that nourish a tumor), and inhibit metastasis (the spread of cancer cells to different sections of the body) [41,42] (Figure 2) (Table 1). Flavonols, including kaempferol, are frequently consumed as glycosides. Studies have demonstrated that glycosides can be absorbed without hydrolysis, despite the suggestion that this requires the prior hydrolysis of absorbable aglycones [31,43,44].

Kaempferol undergoes low to moderate absorption, with the oral bioavailability being very low, estimated at around 2% when compared to intravenous dosages. This limited bioavailability is attributed, at least in part, to extensive first-pass metabolism. Both phase I oxidative metabolism and phase II glucuronidation occur in the intestine as well as in the liver, contributing to the efficient clearance of kaempferol from the systemic circulation upon oral administration [36,45]. In the liver, kaempferol undergoes metabolism, leading to the formation of sulfo-conjugates and glucuronides. Subsequently, in the colon, kaempferol conjugates give rise to phenolic compounds. Ultimately, both kaempferol and the derivatives of kaempferol’s glycosides are excreted in the urine [31,35]. The plasma concentration of kaempferol in rats peaked at 1–1.5 h after oral dose and subsequently dropped up to 6 h. After six hours, no free kaempferol was detected [45]. To address the challenge of low bioavailability associated with kaempferol, research indicates that utilizing nanoformulations such as nanoparticles, nanoemulsions, and nanoencapsulation to deliver kaempferol can significantly enhance its bioavailability and subsequent efficacy. Moreover, these nanoformulations have the potential to enhance selectivity for mutated cells while minimizing their impact on normal cells, thereby offering a promising approach for cancer therapy [46].

**Table 1:** The biochemical and physiological properties of kaempferol.

Properties	Details	References
Molecular Weight	286.24 g/mol	
Classification	Flavonol	[47]
Solubility	Soluble in polar solvents, such as ethanol and methanol	[48]
Melting Point	Approximately 276–278 °C	-
Boiling Point	Decomposes before boiling	-
Color	Yellow crystalline powder	[47]
Odor	Odorless	[47]
Taste	Bitter taste	[47]
UV Absorption	Absorbs UV light at 266 nm and 365 nm	[49]
Biological Sources	Found in tea, apples, onions, grapes, broccoli, and more	[49]
Bioavailability	Moderate absorption in the human digestive system	[50]
Metabolism	Metabolized in the liver, forming various conjugates	[51]
Distribution in Body	Distributed in various tissues	[52]
Half-life in Body	Variable, influenced by factors like age and health	[53]
Excretion	Excreted mainly through urine	[54]
Biological Activities	Antioxidant, anti-inflammatory, anti-cancer, anti-microbial, and neuroprotective properties	[49]
Cellular Mechanisms	Modulates gene expression and signaling pathways such as Nrf2, (PI3K)/AKT, ERK/p38 MAPK, Wnt/β-Catenin.	[38]
Health Benefits	Enhances heart function by reducing myocardial apoptosis, fibrosis, oxidative stress, and inflammation, while maintaining mitochondrial activity and calcium homeostasis. It also provides neuroprotective advantages.	[55,56]
Toxicity	*In vitro* studies have shown that kaempferol is carcinogenic and toxic, while these effects were not reported in *in vivo* screenings.	[35]
Safety	Several *in vitro* studies indicate that kaempferol’s interaction with essential nutrients such as iron and folate may hinder iron bioavailability, absorption, and cellular folic acid uptake. Furthermore, a few *in vitro* studies demonstrate that kaempferol possesses antioxidative properties; excessive supplementation might lead to self-oxidation (pro-oxidation). However, animal studies indicate no pro-oxidation effects after oral consumption. Nevertheless, there have been no human trials investigating the potential toxicity or adverse effects of oral kaempferol consumption.	[57]
Medical Applications	Kaempferol may be used for the therapy of hormone-regulated cancers such as ovarian cancer, breast cancer, cervical cancer, hepatocellular carcinoma, and leukemia.	[49]
Regulatory Status	Considered only as a natural compound, not a regulated drug	-

## 3. Implications of Kaempferol in Gastrointestinal Cancers

**Figure 3:**
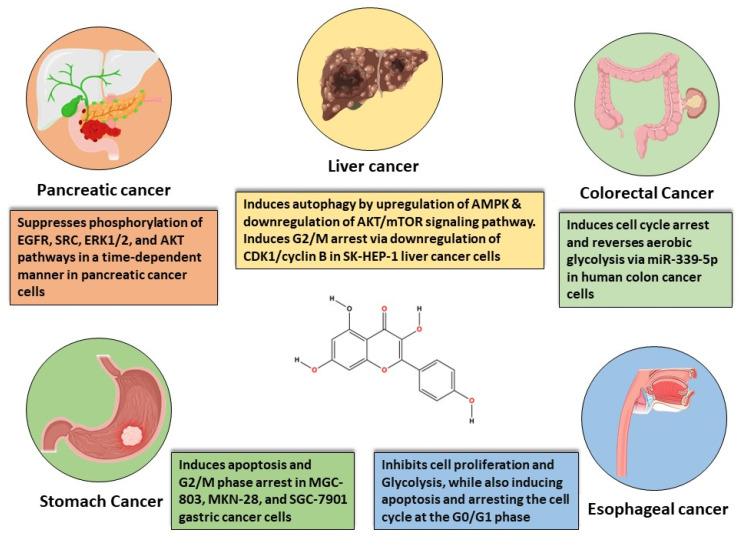
*In vitro*-based anti-cancer mechanisms of kaempferol in various gastrointestinal cancers.

The most common GI cancer malignancies include gastric cancer, colorectal cancer (CRC), liver cancer, and pancreatic cancer. Colorectal cancer is the third most frequent cancer and is associated with a high death rate [58]. On the other hand, stomach cancer ranks fourth in terms of cancer-related mortality [59]. Pancreatic cancer, ranked seventh in terms of global cancer deaths [60], and liver cancer, ranked third in the same category [61], both contribute significantly to cancer-related mortality globally. The following sections explore the most important characteristics of these gastrointestinal tumor malignancies (Figure 3).

### 3.1. Esophageal Cancer

Esophageal cancer is the world’s tenth most frequently occurring malignancy and typically affects men and those born male who are 60 years or older [62]. Esophageal cancer is frequently misdiagnosed until it has progressed to an advanced stage, at which point treatment may involve surgery, radiation, chemotherapy, and supportive care. Recent trends in treating esophageal cancer with kaempferol have shown promising results, with the compound demonstrating potential in inhibiting cell proliferation, inducing apoptosis, and arresting the cell cycle at various phases [26,63].

Kaempferol has been shown to reduce tumor cell growth and impede *in vitro* clonal formation. Additionally, it causes G0/G1 phase arrest in tumor cells [64]. Notably, kaempferol significantly inhibits tumor glycolysis, leading to a marked decrease in glucose uptake and lactate production in tumor cells. This effect is attributed to the downregulation of hexokinase-2, a key enzyme involved in glycolysis. Mechanistic studies have revealed that kaempferol directly affects the activity of the epidermal growth factor receptor (EGFR). The inhibition of EGFR by kaempferol results in the suppression of downstream signaling pathways associated with glycolysis. Further investigations have demonstrated that exogenous overexpression of EGFR in tumor cells attenuates the suppression of glycolysis induced by kaempferol. This suggests that EGFR plays a crucial role in mediating the inhibitory effects of kaempferol on glycolysis in tumor cells. Overall, these findings highlight the potential of kaempferol as a therapeutic agent targeting glycolysis in cancer cells, with EGFR serving as a key molecular target for its anticancer effects [65,66,67].

Mechanistically, it may increase the expression of pro-apoptotic genes such as B-cell lymphoma-2-associated X protein (Bax) via the mitochondrial signaling pathway [68]. It suppresses caspase-9 expression while also downregulating the production of the anti-apoptotic protein B-cell lymphoma-2 (Bcl-2). Caspase-9 inhibition activates caspase-3, resulting in a caspase cascade that leads to apoptosis [67,69]

Furthermore, kaempferol has potential in the therapy of human esophageal cancer cell lines. Flavone treatment can cause G2/M arrest by increasing GADD45β and 14-3-3ε levels, while decreasing cyclin B1 mRNA and protein. It can also promote p53-independent mitochondrial apoptosis by upregulating PIG3 and promoting caspase-9 and caspase-3 cleavage [70]. These findings emphasize kaempferol’s therapeutic potential for esophageal cancer. The chemical has been proven to be more effective than docetaxel in reducing primary tumor growth, and it can overcome cisplatin resistance [26]. Even with these encouraging results, more research is required to fully understand the safety and effectiveness of kaempferol in the treatment of esophageal cancer. This research should include clinical trials as well as long-term follow-up studies.

### 3.2. Gastric Cancer

As per the GLOBOCAN 2022 statistics, stomach cancer had approximately 1,089,103 incidents and caused 768,793 deaths, which makes it the fourth most common cause of death globally [59]. East Asia, Eastern Europe, and South America are among the regions with higher incidence and fatality rates. Males are noticeably twice as likely than females to have stomach cancer. Ageing populations and better living circumstances have an impact on the prevalence of *Helicobacter pylori*, a significant cause of stomach cancer, even though incidence and death rates have gradually declined over the past century [71].

Researchers have extensively examined the impact of kaempferol on various cells of gastric cancer. In a 2010 study, kaempferol demonstrated a dose- and time-dependent induction of apoptosis and G2/M phase arrest in MGC-803 cells [61]. Another study revealed that kaempferol induced apoptosis in MKN-28 gastric cancer cell lines, while showing no effect on conventional gastric epithelial cell lines (GSE-1) [28]. Notably, xenograft tumor development was reduced without affecting body weight, the liver, or spleen in kaempferol-treated mice [28]. Additionally, SGC-7901 and MKN-28 cell lines exhibited lower levels of p-ERK, p-Akt, and COX-2 expression [32]. In 2018, researchers investigated the molecular processes and biological activity of kaempferol in stomach cancer therapy. According to the study, kaempferol induced autophagy and cell death, lowered p62 production, and accelerated the transition from LC3-I to LC3-II through an IRE1-JNK1-mediated Bcl-2-Beclin-1 link [72]. The research also uncovered another mechanism involving the HDAC/G9a pathway for kaempferol-mediated epigenetic alterations related to autophagy and cell death [72].

### 3.3. Colorectal Cancer

CRC stands out as one of the most prominent solid tumors in the Western world. The development of abnormal growths known as polyps in the colon or rectum is a precursor to colorectal cancer, with the potential for these polyps to progress into cancer over time [73]. Symptoms of CRC encompass alterations in bowel habits, the presence of blood in the stool, and abdominal pain [74]. Based on the molecular mechanisms of tumor formation and progression, CRC is generally classified into three classes: sporadic, inflammation-dependent, and familial [75,76]. Of these, sporadic CRC has the highest prevalence, approximately 75% [77]. In patients with inflammatory bowel disease (IBD), chronic inflammation plays a major role in the carcinogenesis of inflammation-dependent CRC [78,79]. Treatment options of CRC are determined on the stage of the disease, the patient’s performance level, and, increasingly, the molecular makeup of the tumor. Because malignancies are diagnosed at earlier stages in countries with monitoring programs, both the incidence and fatality rates have decreased [27,80]. Colorectal cancer therapy options are consistent in the metastatic scenario. Over time, CRC treatment techniques have evolved from using 5-fluorouracil (5-FU) as a single agent to more complex combination regimens that include 5-FU together with oxaliplatin, irinotecan, or a combination of both [27,80]. While traditional treatment approaches including radiation, chemotherapy, and surgery have a significant impact on the management of colorectal cancer, drug resistance and toxicity continue to be major obstacles. As a result, dietary therapy agents, especially natural products, may have been thought of as the safest substitutes for treating CRC, improving the symptoms and quality of life for individuals with the disease [81].

Kaempferol has been recommended as a promising drug for the prevention of colon cancer due to its capacity to cause cell cycle arrest, especially in HT-29 human colon cancer cells [82]. It has been shown to overcome colorectal LS174 cancer cell resistance to 5-Fu via decreasing PKM2-mediated glycolysis, implying that kaempferol may have a chemotherapeutic role, either alone or in conjunction with 5-Fu [27,80]. Kaempferol’s antioxidative and anti-inflammatory properties may help to regulate cancer growth and progression [25]. Additionally, it has been demonstrated to reverse aerobic glycolysis through regulation mediated by miR-339-5p, with potent anti-colon cancer effects [83]. Regarding how it works, kaempferol encourages colon cancer cells to express microRNA-326 (miR-326). Furthermore, miR-326 could obstruct PKM mRNA’s alternative splicing factors indirectly. As a result, this modulation aids in reversing the colorectal cancer cells’ resistance to 5-Fu.

### 3.4. Pancreatic Cancer

Pancreatic cancer (PC) patients show no noticeable symptoms until the disease reaches an advanced stage characterized by invasive pancreatic metastasis, making early detection challenging. Consequently, pancreatic cancer has emerged as one of the most challenging cancer malignancies [84,85]. A significant proportion of patients experience relapse, and despite potentially aggressive treatments, the 5-year survival rate remains low, ranging from 2% to 9% [70]. Pancreatic ductal adenocarcinoma is the most frequent pancreatic cancer. Family history, pancreatitis, and diabetes are all risk factors for pancreatic cancer [84].

Most PC malignancies are classified as ductal adenocarcinoma and hence represent exocrine pancreatic malignancy, whereas a minority are neuroendocrine tumors. The bulk of pancreatic ductal adenocarcinomas originate from precursor lesions classified as pancreatic intraepithelial neoplasia, which evolve in a stepwise manner through the acquisition of genetic changes to result in the formation of overt pancreatic ductal adenocarcinoma [86].

**Figure 4:**
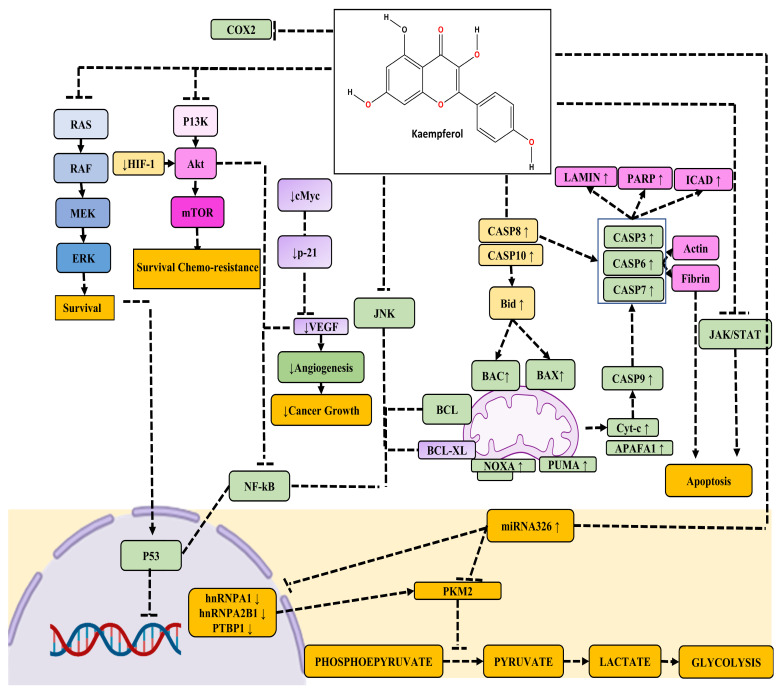
The figure illustrates the anti-cancer activity of kaempferol across various molecular pathways. Kaempferol exerts its anti-cancer effects through intricate signaling pathways. It inhibits the MAPK, PI3/AKT/mTOR, and JNK pathways, thereby suppressing the NF-κB pathway. Simultaneously, the downregulation of the JNK pathway reduces the expression of anti-apoptotic proteins Bcl-XL and Bcl-2. Moreover, kaempferol enhances the expression of pro-apoptotic factors such as CASP8 and CASP10, leading to an increase in mitochondrial proteins BAX and BAK, and the activation of CASP8, CASP3, CASP6, and CASP7, ultimately resulting in apoptosis. Additionally, kaempferol promotes the expression of key apoptotic regulators including Cytochrome c, Apaf-1, NOXA, and PUMA, which are pivotal in orchestrating the apoptotic cascade. Notably, the inhibition of the JNK pathway by kaempferol has a dual impact on apoptosis, as it dampens P53-mediated cell apoptosis while simultaneously boosting CASP-mediated apoptosis, thereby contributing to the overall anti-cancer properties of kaempferol.

Kaempferol suppresses PC cell proliferation and migration by modulating the EGFR-related pathway. Its effective, low-dose migratory activity reduction in human pancreatic cancer cells without causing cellular harm is very notable [87]. Kaempferol reduces PC cell viability by promoting apoptosis. Kaempferol’s anti-cancer action is mediated by the suppression of phosphorylation levels in EGFR, ERK1/2, Src, and AKT pathways demonstrated in a time-dependent manner in Miapaca-2 and Panc-1 cells [87,88,89]. Furthermore, in the case of PC, the promotion of apoptosis is mediated by AKT/mTOR signaling and TGM2 pathways (Figure 4).

Research on kaempferol indicates that its anti-cancer effects on PC cell development may stem from its cytotoxic and antioxidative activities [90,91]. Kaempferol has been observed to inhibit cell growth by inducing apoptosis in cancer cells, notably through the downregulation of proliferating cell nuclear antigen (PCNA) and activation of cleaved caspase-3 pathways. This effect was dose-dependent, as evidenced by the varied responses observed at different dosages of kaempferol. Interestingly, when pancreatic cancer cells were pre-treated with z-VAD-FMK, an inhibitor of caspase activity, the inhibitory effect of kaempferol on cell growth was significantly reduced, suggesting the involvement of caspase-dependent pathways in its mechanism of action [92]. Additionally, kaempferol treatment suppresses the formation of fatty acids (FAS) and the growth of cells, causing the cells in breast and prostate cancer to undergo apoptosis. This demonstrates that blocking FAS could be linked to apoptosis in a range of cancer cells [93]. These findings show that kaempferol can function as a safe chemotherapeutic reagent in human PC cells, making it a promising option for PC treatment [88].

### 3.5. Liver Cancer

The most common form of liver cancer is called hepatocellular carcinoma (HCC), which starts in the main liver cell type called hepatocytes [61]. Liver cancer may be classified into two main categories: primary liver cancer, which starts inside the liver, and secondary liver cancer, which develops when cancer cells migrate to the liver from another area of the body. A hard mass just below the rib cage on the right side, upper abdominal pain, swollen belly, pain in the right shoulder blade or back, and jaundice are typical signs of liver cancer [94,95].

Mylonis et al. discovered that in hypoxic environments, kaempferol reduces the survival of hepatoma (Huh-7) cancer cells, hypoxia-inducible factor 1 (HIF-1), and mitogen-activated protein kinase (MAPK) [96]. The mitochondrial tricarboxylic acid (TCA) cycle, membrane-bound ATPase (Ca^2+^, ATPase, Na^+^/K^+^ ATPase, Mg^2+^, and ATPase), nucleic acids, and multiple classes of enzymes like G-6-P, F-1,6-diphosphatase and hexokinase, which includes carbohydrate-metabolizing enzyme classes, were significantly modulated by kaempferol in an *in vivo* study. This modification was seen in HCC caused by aflatoxin B1 (AFB1), demonstrating strong anti-carcinogenic qualities [97]. Another investigation identified kaempferol as an anti-inflammatory agent, demonstrating its capacity to dose-dependently inhibit the downregulation of tumor necrosis factor-alpha (TNF-α) on liver-X-receptor alpha (LXR-α) [98]. Kaempferol has been shown to exert hepatoprotective effects by modulating key enzymes and signaling pathways in the liver. In pre-clinical studies, kaempferol demonstrated hepatoprotective effects by the activation of SIRT1 and downregulation of CYP2E1and PARP1. However, the exact mechanism by which kaempferol inhibits CYP2E1 after treatment still needs to be explored [99].

Kaempferol has been found to induce apoptosis and provoke G2 m stage cell cycle arrest, consequently impeding the invasion and migration of cancerous cells. This effect is mediated through various mechanisms. Kaempferol triggers the release of cytochrome-c by generating ROS, which in turn initiates mitochondria swelling and loss of mitochondrial membrane potential (MtMP), ultimately leading to increased levels of caspase 3. Furthermore, Kaempferol has been observed to upregulate the expression of several key proteins involved in cellular signaling pathways. These include non-receptor tyrosine-protein kinase (TYK-2), Janus kinase-1 (JAK-1), microtubule-associated protein-1A-1B light chain-3 (MAPILC3), STAT1-2, and autophagy genes (Atg 5, Atg 7, and Atg 12), along with beclin-1 and phosphatase and tensin homolog (PTEN). Kaempferol downregulates the expression of cytokine signaling-3 (SOCS-3), PI3K-AKT-mTOR, miRNA-21, signal transducer and activator of transcription-3 (STAT-3), phosphorylated-mTOR signaling pathways, and HIF-1 in HCC. Overall, these findings highlight the multifaceted effects of kaempferol in modulating cellular pathways involved in cancer progression, underscoring its potential as a therapeutic agent in the treatment of HCC [96,100,101].

Additionally, kaempferol has been shown to decrease TNF-α production by inhibiting IκB kinase (IKK) and MAPK, thereby suppressing NF-κB activity and its associated pathway. However, when faced with the inflammatory conditions induced by TNF-α stimulation, kaempferol did not fully restore ABCA1 expression back to untreated levels, although it did mitigate the extent of expression. Interestingly, ABCA1 mRNA expression was significantly increased with a high dose of kaempferol alone, indicating a positive correlation between ABCA1 and kaempferol. This implies that kaempferol may be more beneficial as a preventive agent [102]. Kaempferol has been shown in several studies to protect the liver against a variety of oxidative stressors [103,104,105]. Furthermore, data demonstrated that kaempferol had no substantial toxicity on normal cells. kaempferol’s multi-targeting action can minimize medication resistance throughout treatment [106].

## 4. Synergistic Effect and the Significance of Nanotechnology in Kaempferol’s Therapeutic Efficacy

Nanotechnology has emerged as a crucial tool in both the diagnosis and therapy of gastrointestinal (GI) cancers, which rank among the most prevalent and lethal cancers worldwide. Nanoparticles offer numerous advantages over conventional cancer treatments, including heightened specificity in detection, reduced drug toxicity, and enhanced capabilities in therapy for GI cancers [107]. Various types of nanoparticles are employed in the treatment of GI cancers, encompassing quantum dots (QDs), carbon nanotubes (CNTs), metallic nanoparticles (MNPs), and dendrimers. These nanoparticles exhibit distinct optical properties, excellent biocompatibility, surface effects, and small size effects, rendering them well suited for both diagnostic and therapeutic purposes [108]. Among these, metallic nanoparticles, such as gold and iron oxide nanoparticles, have garnered significant attention due to their promising properties and therapeutic potential in cancer treatment. Gold nanoparticles (GNPs), for instance, are established nanostructures known for their strong light absorption, enabling them to generate thermal energy that facilitates the photothermal destruction of cancerous tissue. This photothermal ablation can be achieved through various means, including GNPs, selenium nanoparticles, or copper sulfide nanoparticles [109]. Additionally, nanogels represent another type of nanoparticle utilized in GI cancer treatment. These nanoscale networks, formed through either noncovalent interactions or covalent crosslinking of polymer chains, offer distinct advantages. Nanogels are particularly noteworthy as oral drug delivery systems due to their heightened sensitivity to external stimuli compared to other delivery systems, making them promising candidates for effective GI cancer therapy [107].

Nanotechnology has been useful in the chemotherapy of malignant GI tumors because of its ability to target specific enzymes and the tumor’s unique microenvironment. The pH changes between tissue sites, as well as the particular hypoxic conditions found in malignant GI tumors, provide guidance for the development of responsive nanocarriers. Enzyme reactions can be exploited in nanodrug delivery systems, such Gal-Dox, which targets β-galactosidase and has substantial anticancer effects [89]. Another application of nanotechnology in GI cancers is drug or gene delivery systems. Nanodevices can load drugs at a high concentration, which are efficiently delivered to specific sites with fewer side effects. Due to their high sensitivity, specificity, and permeability, nanotechnologies have mostly been used in MRI-based clinical applications for the imaging of the GI system and tumor detection. Drug delivery based on nanotechnology will be important for future medical care, particularly cancer treatment. Nanomaterials exhibit remarkable use for enhancing therapeutic effectiveness due to their high biocompatibility [107,110].

Research investigating the potential synergy between kaempferol and nanotechnology in the treatment of GI cancer has received attention. Kaempferol’s limited bioavailability has been a concern, but studies using nanoformulations have suggested an enhancement in its efficacy. For instance, kaempferol-coated silver nanoparticles (AgNPs) exhibited a synergistic impact on apoptosis in HepG2 cells. A reduction in Bcl-2 levels, a rise in Bax and Cyc-c levels, the activation of caspase-3 (due to mitochondrial membrane rupture), and an improvement in p53-mediated cell cycle arrest were the indicators of this impact. According to the study, kaempferol-coated AgNPs may induce oxidative stress-mediated apoptosis and cell cycle arrest in liver cancer (HepG2) cells, hence having an anti-cancer impact [111]. It has been demonstrated that kaempferol-conjugated gold nanoclusters can target and eliminate the nucleus of cancer cells. Nanoformulations incorporating kaempferol have shown significant cancer cell inhibition activities by reducing cell viability [112,113], inducing apoptosis [114], damaging cancer cell nuclei [112], and increasing LDH leakage percentage [111]. PEGylated AuNPs-DOX@ kaempferol showed significant anticancer efficacy *in vivo*, leading to a decrease in tumor volume [94]. These findings suggest that kaempferol-conjugated gold nanoclusters hold potential for the development of effective cancer treatments.

Kaempferol conjugation with nanoparticles has been extensively studied for combating cancer, showing promising results in various cancer types (Table 2). However, there is a lack of research specifically focused on GI cancers, highlighting the need for further exploration in this area.

## 5. Safety Aspects of Kaempferol

Kaempferol has been proven to have low toxicity in various investigations, including in bladder cancer, where it was discovered to be a strong inhibitor with good safety [26]. Kaempferol inhibits malignant cells while not affecting healthy ones, potentially contributing to its safety profile [122]. Its effects on cancer cells are dose-dependent. Higher doses may have a greater impact on cancer cells while limiting the impact on healthy cells. Synergistic effects of kaempferol with other anti-cancer medications can improve safety by reducing individual doses [83,122]. Nano-based formulations of kaempferol have been produced to overpower the quick degradation and decrease in toxicity, which may further improve its safety profile [115]. Indeed, even if the results are encouraging, more investigation is required to fully comprehend the safety concerns associated with kaempferol use in the prevention of GI malignancies. Comprehensive *in vivo* investigations and well-planned clinical trials are needed to evaluate the safety and effectiveness of kaempferol in cancer prevention.

## 6. Conclusions

Kaempferol is a bioflavonoid molecule that helps to reduce the risk of hormone-related malignancies. This compound’s principal actions include oncogene-induced apoptosis and growth inhibition, but it is also thought to increase the host’s immune response. When kaempferol is present in larger amounts, it has an anti-cancer effect, but when it is present in smaller amounts, it has a pro-cancer function. Kaempferol has various problems in health management, including quick metabolism, low water solubility, lysosomal breakdown, and efficient elimination from the body. Not much study has been undertaken on kaempferol’s possible involvement in cancer treatment due to its limited bioavailability. Nanotechnology-based formulations have recently been produced, and their promise for cancer treatment has been established through *in vitro* testing. However, more *in vivo* study is needed to improve kaempferol’s bioavailability, allowing for the investigation of its involvement in cancer management with a specific focus on tumor cells. Furthermore, kaempferol has synergistic effects with anti-cancer drugs, increasing efficacy by inhibiting and activating gene activity. While a few human-based studies have been undertaken to assess its relevance in health management, there is a need to move the importance of kaempferol from preclinical to clinical cancer therapy approaches. This transition will provide a better understanding of kaempferol’s potential in cancer treatment and health management.

## Figures and Tables

**Table 2:** List of nanoparticles conjugated with kaempferol in cancer.

S. No.	Conjugated Nanoparticles	Composition of Nanoparticle	Cancer Type (*In Vitro/Vivo* Model)	Outcome	Ref.
1	PEO-PPO-PEO nanoparticles	poly(ethylene oxide) -poly(propylene oxide) -poly(ethylene oxide)	Ovarian (OVCAR-3)	Cancer cells were inhibited but normal cell vitality was lowered as well	[115]
2	PLGA nanoparticles	Poly(DL-lactic acid-co-glycolic	Ovarian (OVCAR-3)	Effectively reduced cancer cell viability	[115]
3	Nanostructured Lipid carriers	-------	Glioblastoma Multiforme (U-87 MG)	Elevated toxicity against cancer cells	[116]
4	Kf-CS/Ag nanocomposite	Chitosan/silver Nanocomposite	Breast (MDA MB-231)	Exhibited significant inhibitory effects on *in vitro* and apoptotic cell death	[117]
5		Nanomaterial (PEGylated AuNPs-DOX @kaempeferol)	Colon HT-29 & mice)	Reduction in tumor volume in mice	[118]
6	CNPsLE	*A Judaica* extract and chitosan nanoparticle- loaded extract	Prostate (PC3)	CNPsLE showed enhanced selective toxicity (IC_50_: 20.8 µg/mL) outperforming the extract (IC_50_: 76.09 µg/mL)	[119]
7	K-AuNCs	Kaempferol-conjugated gold nanocluster	Lung (A549)	Nanocluster exhibited lower toxicity to normal cells and higher toxicity to cancer cells	[112]
8	M@CaCO3@KAE	KAE loaded into CaCo3 nanoparticles incorporated with the cell membrane	-------	It responds to the tumor microenvironment, releasing KAE and calcium ions; damages mitochondrial cytoskeleton collapse, oxidative stress, apoptosis, *in vivo* tumor inhibition	[120]
9	KPF-MNE	KPF-loaded mucoadhesive nanoemulsion	Glioma (C6; *in vivo*)	Reduces cancer cell viability via induction of apoptosis	[121]
10	AgNPs	Kaempferol-coated silver nanoparticles	Liver (HepG2)	Decreases viability of cancer cells	[111]

## Data Availability

Data sharing is not applicable to this article as no datasets were generated or analyzed during this current study.
